# A Defensin from the Model Beetle *Tribolium castaneum* Acts Synergistically with Telavancin and Daptomycin against Multidrug Resistant *Staphylococcus aureus*

**DOI:** 10.1371/journal.pone.0128576

**Published:** 2015-06-10

**Authors:** Rajmohan Rajamuthiah, Elamparithi Jayamani, Annie L. Conery, Beth Burgwyn Fuchs, Wooseong Kim, Tatiana Johnston, Andreas Vilcinskas, Frederick M. Ausubel, Eleftherios Mylonakis

**Affiliations:** 1 Division of Infectious Diseases, Rhode Island Hospital, Alpert Medical School of Brown University, Providence, RI, 02903, United States of America; 2 Massachusetts General Hospital, Harvard Medical School, Boston, MA, 02114, United States of America; 3 Institute of Phytopathology and Applied Zoology, Justus-Liebig University, Heinrich-Buff-Ring 26–32, 35392, Giessen, Germany; Nanyang Technological University, SINGAPORE

## Abstract

The red flour beetle *Tribolium castaneum* is a common insect pest and has been established as a model beetle to study insect development and immunity. This study demonstrates that defensin 1 from *T*. *castaneum* displays *in vitro* and *in vivo* antimicrobial activity against drug resistant *Staphylococcus aureus* strains. The minimum inhibitory concentration (MIC) of defensin 1 against 11 reference and clinical staphylococcal isolates was between 16–64 μg/ml. The putative mode of action of the defensin peptide is disruption of the bacterial cell membrane. The antibacterial activity of defensin 1 was attenuated by salt concentrations of 1.56 mM and 25 mM for NaCl and CaCl_2_ respectively. Treatment of defensin 1 with the reducing agent dithiothreitol (DTT) at concentrations 1.56 to 3.13 mM abolished the antimicrobial activity of the peptide. In the presence of subinhibitory concentrations of antibiotics that also target the bacterial cell envelope such as telavancin and daptomycin, the MIC of the peptide was as low as 1 μg/ml. Moreover, when tested against an *S*. *aureus* strain that was defective in D-alanylation of the cell wall, the MIC of the peptide was 0.5 μg/ml. Defensin 1 exhibited no toxicity against human erythrocytes even at 400 μg/ml. The *in vivo* activity of the peptide was validated in a *Caenorhabditis elegans*-MRSA liquid infection assay. These results suggest that defensin 1 behaves similarly to other cationic AMPs in its mode of action against *S*. *aureus* and that the activity of the peptide can be enhanced in combination with other antibiotics with similar modes of action or with compounds that have the ability to decrease D-alanylation of the bacterial cell wall.

## Introduction

Antimicrobial peptides (AMPs) are short peptides less than 50 amino acids that are naturally produced in diverse organisms including plants and metazoans in response to pathogen insult [1]. Cationic AMPs are a subcategory of AMPs that are rich in cationic and hydrophobic residues, giving them an overall net positive charge, which in turn enables the AMPs to bind and disrupt the integrity of the negatively charged bacterial cell envelope. In addition to causing membrane disruption, AMPs have also been demonstrated to possess immunomodulatory properties [2]. AMPs have been underexploited as potential therapeutics in the past, but the rising tide of drug resistance in *Staphylococcus aureus* and other bacterial pathogens, coupled with the shrinking pool of available drugs for treating these infections, has necessitated the need to develop alternative treatment strategies and therapeutic compounds. As a consequence, the use of AMPs is making a resurgence [1].

*Staphylococcus aureus* has emerged as a leading cause of hospital and community-acquired infections [3]. Although vancomycin is currently used to treat MRSA as an antibiotic of last resort, vancomycin-resistant *S*. *aureus* (VRSA) strains have started to emerge, motivating the urgent development of new antibiotics effective against antibiotic-resistant *S*. *aureus*. Overall, *S*. *aureus* causes approximately 10,800 deaths per year in the United States and approximately 50% of these are due to MRSA. These statistics underscore the urgent need for novel anti-infectives effective against *S*. *aureus*.

In the initial part of this study we screened a set of 65 insect-derived AMPs for activity against methicillin resistant *S*. *aureus* (MRSA). Only one of the 65 AMPs, defensin 1 from the model beetle *Tribolium castaneum* (Def1 Genbank accession number: XM_968482) exhibited promising activity against MRSA. The genome of this beetle encodes three defensins, which have been demonstrated to be induced upon septic injury [4]. The aim of this study was to elucidate the mode of action of defensin 1 from *Tribolium* and to explore whether it displays combinatorial activity with antiobiotics and to test its toxicity against mammalian cells as well as its *in vivo* efficacy.

## Materials and Methods

### Bacterial and nematode strains

The staphylococcal strains tested in this study are listed in Table 1. All strains were grown at 37°C in tryptic soy broth (TSB, Becton Dickinson and Company, NJ, USA). The following bacterial strains were also tested for sensitivty to AMPs: *Enterococcus faecium* (E007), *Klebsiella pneumoniae* (ATCC 77326), *Acinetobacter baumannii* (ATCC 17978), *Pseudomonas aeruginosa* (PA14) and *Enterobacter aerogenes* (EAE 2625). The *Caenorhabditis elegans glp-4(bn2);sek-1(km4)* double mutant strain was maintained at 15°C on a lawn of *Escherichia coli* strain HB101 on 10 cm plates [5]. The *glp-4(bn2)* mutation renders the strain incapable of producing progeny at 25°C [6] and the *sek-1(km4)* mutation enhances sensitivity to various pathogens [7], thereby reducing assay time.

**Table 1:** Comparative antibacterial activities of defensin 1, vancomycin, oxacillin, and mupirocin against staphylococcal strains.

Strain	μg/ml			
	Defensin 1	Vancomycin	Oxacillin	Mupirocin
MW2	64	2	64	< = 0.125
USA100 [28]	64	2	< = 0.0625	0.125
USA300 [28]	64	4	>64	0.125
USA400 [28]	16	2	>64	0.25
RN4220 [29]	64	2	< = 0.125	< = 0.0625
Newman [30]	64	2	< = 0.0625	0.25
Newman *∆dltA* [15]	0.5	ND	ND	ND
BF1*	32	2	>64	0.125
BF5*	32	2	>64	0.125
BF7*	32	2	>64	0.125
BF8*	32	2	>64	0.25
BF9*	32	2	0.25	0.125
BF10*	32	2	>64	0.125
BF11*	32	2	>64	0.125
*S*. *epidermidis* (9142) [31]	64	4	>64	< = 0.0625

* indicates *S*. *aureus* clinical isolates.

### Synthesis of insect-derived antimicrobial peptides

Sixty five insect AMPs were synthesized based on publicly available sequence data or sequences generated by transcriptomic analyses of immune-challenged insects, such as *T*. *castaneum*, medicinal maggots of the blow fly *Lucilia sericata* [8], the invasive harlequin ladybird *Harmonia axyridis* [9], and the greater wax moth *Galleria mellonella* [10]. These 65 peptides (S1 Table) were produced by solid-phase synthesis (commissioned work by Panatecs GmbH, Tübingen Germany). Confirmation of correct formation of disulfide linkages in synthetic defensin 1 was performed by mass spectrometric analysis of the fragment spectra of disulfide like tryptic peptides (data not shown). The 65 peptides were extracted to ≥80% purity by reverse-phase chromatography by GenScript (NJ, USA). Following the initial screening, synthetic *Tribolium* defensin 1 was resynthesized and processed to more than 95% purity, prior to further investigations.

### Antimicrobial susceptibility testing

Antimicrobial susceptibility assays were performed in 96 well plates in triplicate using Müller-Hinton broth (Becton Dickinson and Company, NJ, USA) with an assay volume of 100 μl. The 65 AMPs were arrayed in 96 well microplates to a final concentration of 100 μg/ml. The MRSA strain MW2 was grown overnight in TSB and the bacterial inoculum was added to the assay plate at an adjusted initial OD_600_ of 0.03. After overnight incubation at 35°C, the absorbance of each well was measured to determine antimicrobial activity. The procedure for determining the minimum inhibitory concentration (MIC) was adapted from an earlier protocol [11]. In brief, two-fold serial dilutions were carried out to get the test compounds in the concentration range 0.0625–64 μg/ml. After overnight incubation at 35°C, the absorbance was measured to determine antimicrobial activity.

### Checkerboard assay for combining antimicrobials

The antimicrobial activity of a combination of defensin 1 with either telavancin or daptomycin was determined through a checkerboard assay described in [11]. Briefly, the compounds whose combinations were being tested were arrayed in serial concentrations, vertically for one compound and horizantally for the other compound in the same 96 well microplate. The rest of the procedure involving addition of bacteria and measurement of growth was carried out as described in the previous section for measurement of MIC.

The Fractional Inhibitory Concentration (FIC) index for two compounds A and B is defined by the following equation: FIC = ((A/MIC^A^) + (B/MIC^B^)). MIC^A^ and MIC^B^ are MICs of compound A or B respectively. (A) is the lowest concentration of compound A in combination with compound B that inhibits bacterial growth and (B) is the viceversa. An FIC <0.5 indicates synergism between the compounds being tested.

### Bacterial cell membrane permeabilization assay

Permeabilization of bacterial membranes was determined by Sytox Green (Life Technologies, CA, USA) uptake by bacterial cells in 96 well plates (CLS3300, Corning, NJ, USA), performed in triplicate. Logarithmically growing MRSA strain MW2 cells were harvested by centrifugation at 4000 rpm for 5 minutes, the pellet was washed twice in PBS and resuspended in PBS to an absorbance of 0.5 at 595 nm. Sytox Green was added to the cells at a final concentration of 5 μM and incubated in the dark for 30 minutes, followed by addition of the cells to compounds serially diluted in PBS. The fluorescence intensities were measured at different time points (excitation = 485 nm, emission = 530 nm).

### Human blood hemolysis

The protocol to test the ability of compounds to cause hemolysis of human erythrocytes (Rockland Immunochemicals, PA, USA) was adapted from Rosch et al. [12]. In a 96 well plate, 50 μl of 2% human erythrocytes suspended in PBS was added to 50 μl of compounds serially diluted in PBS and incubated at 37°C for 1 hour. The plate was then centrifuged at 500 G for 5 minutes and 50 μl of the supernatant from each well of the assay plate was transferred to a fresh 96 well plate. Hemolysis was confirmed by both visual observation and measuring absorbance at 540 nm. Experiments were performed in triplicate.

### *C*. *elegans*-MRSA liquid infection assay

The *C*. *elegans*-MRSA liquid infection assay has been described [5]. In brief, the infection assay was performed in standard 384-well assay plates with the test compounds or 1% DMSO as control. *S*. *aureus* MW2 was added to the wells at a final OD_600_ of 0.04, followed by automated transfer of 15 adult *glp-4(bn2);sek-1(km4)* worms to each well of the assay plate using a COPAS large particle sorter (Union Biometrica, MA, USA). After 5 days of incubation at 25°C, the worms were stained with the vital dye Sytox Orange after washing away the bacteria. The assay plates were imaged using an Image-Xpress Micro automated microscope (Molecular Devices, CA, USA), capturing both transmitted light and TRITC (535 nm excitation, 610 nm emission) fluorescent images with a 2X objective. The images from the infection assay were processed using the analysis software CellProfiler (http://www.cellprofiler.org/) and the percentage worm survival was calculated for each well of the assay plates. The entire assay was performed in duplicate.

## Results

### Defensin 1 from *T*. *castaneum* inhibits growth of *S*. *aureus*

**Fig 1:**
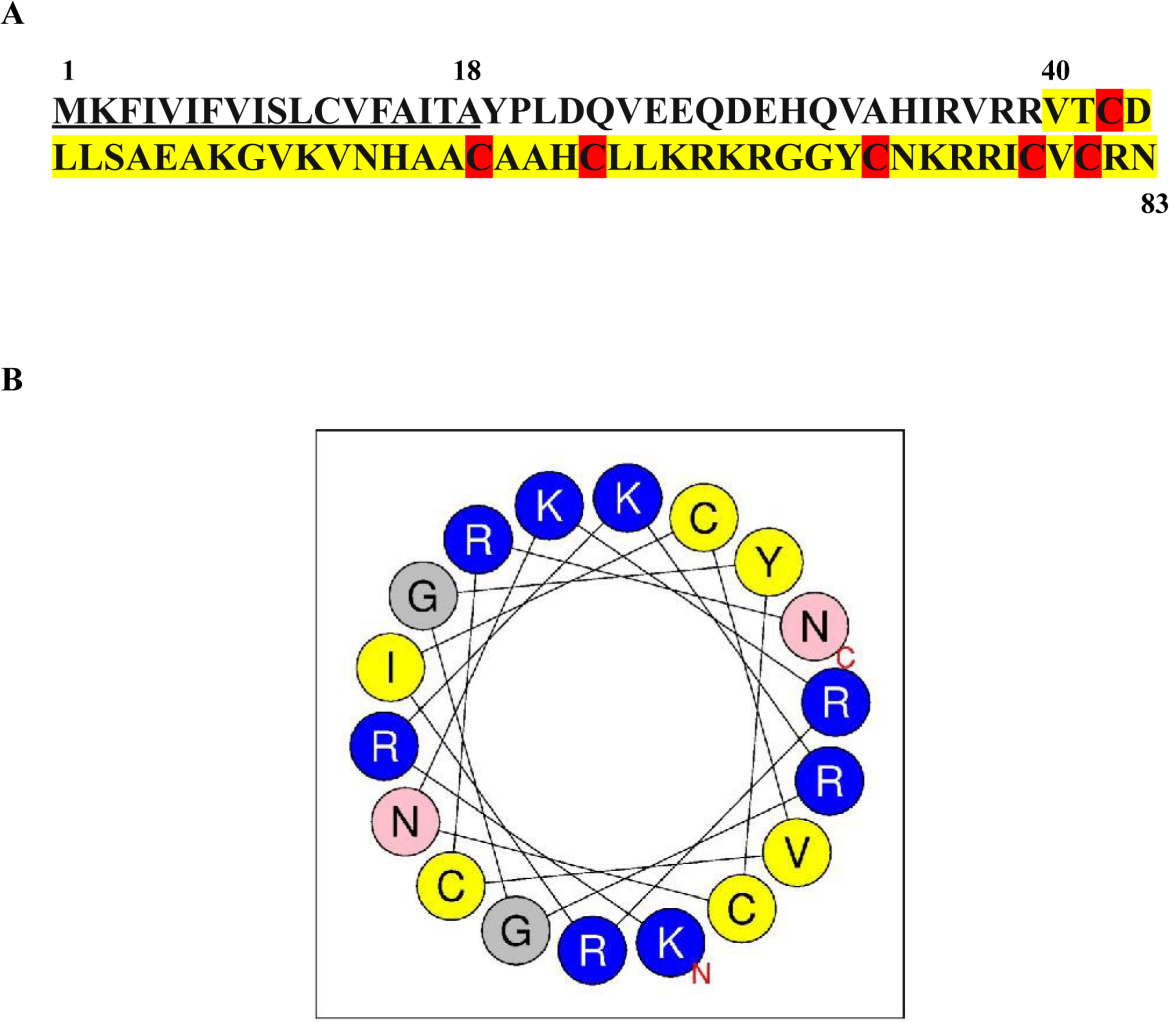
Amino acid sequence of defensin 1. **(A)** The predicted full length of the peptide is 83 a.a, the underlined region is the signal peptide cleavage site (www.predisi.de), the region highlighted in yellow represents the mature peptide sequence tested in this study. The cysteine residues that are conserved among the arthropod defensin family are highlighted in red. **(B)** Helical wheel diagram of residues between 66–83 of defensin 1 determined by the Heliquest method. Blue spheres indicate positively charged residues.

A set of 65 AMPs (S1 Table) synthesized *in vitro* based on genomic sequences from a variety of insects was tested for inhibition of growth of MRSA strain MW2 in a broth assay at a final AMP concentration of 100 μg/ml. Among the 65 AMPs only defensin 1 from *Tribolium castaneum* (Fig 1A), inhibited the growth of MW2 cells and the rest of the AMPs behaved similar to the DMSO control and did not restrict bacterial growth as determined by visual and spectrophotometric observation (S1 Table).

Defensin 1 was retested for antimicrobial activity by determining its MIC against staphylococci of diverse genetic backgrounds, including MW2, USA100, USA300, USA400, RN4220, Newman and *Staphylococcus epidermis* 9142. We also compared the anti-staphylococcal activity of defensin 1 with well known commercially available anti-staphylococcal drugs such as vancomycin, oxacillin and mupirocin. Defensin 1 displayed MICs between 16–64 μg/ml against the tested strains (Table 1). The antimicrobial activity of defensin 1 was also evaluated against 7 clinical staphylococcal isolates collected by our laboratory (BF 1, 5, 7–11). Among these isolates; BF 1, 5, 7, 8, 10 and 11 demonstrated MICs to oxacillin of >64 μg/ml, whereas defensin 1 displayed an MIC of 32 μg/ml against all tested clinical isolates (Table 1).

### The antimicrobial activity of defensin 1 is modulated by cations and D-alanylation of bacterial wall teichoic acids (WTAs)

**Fig 2:**
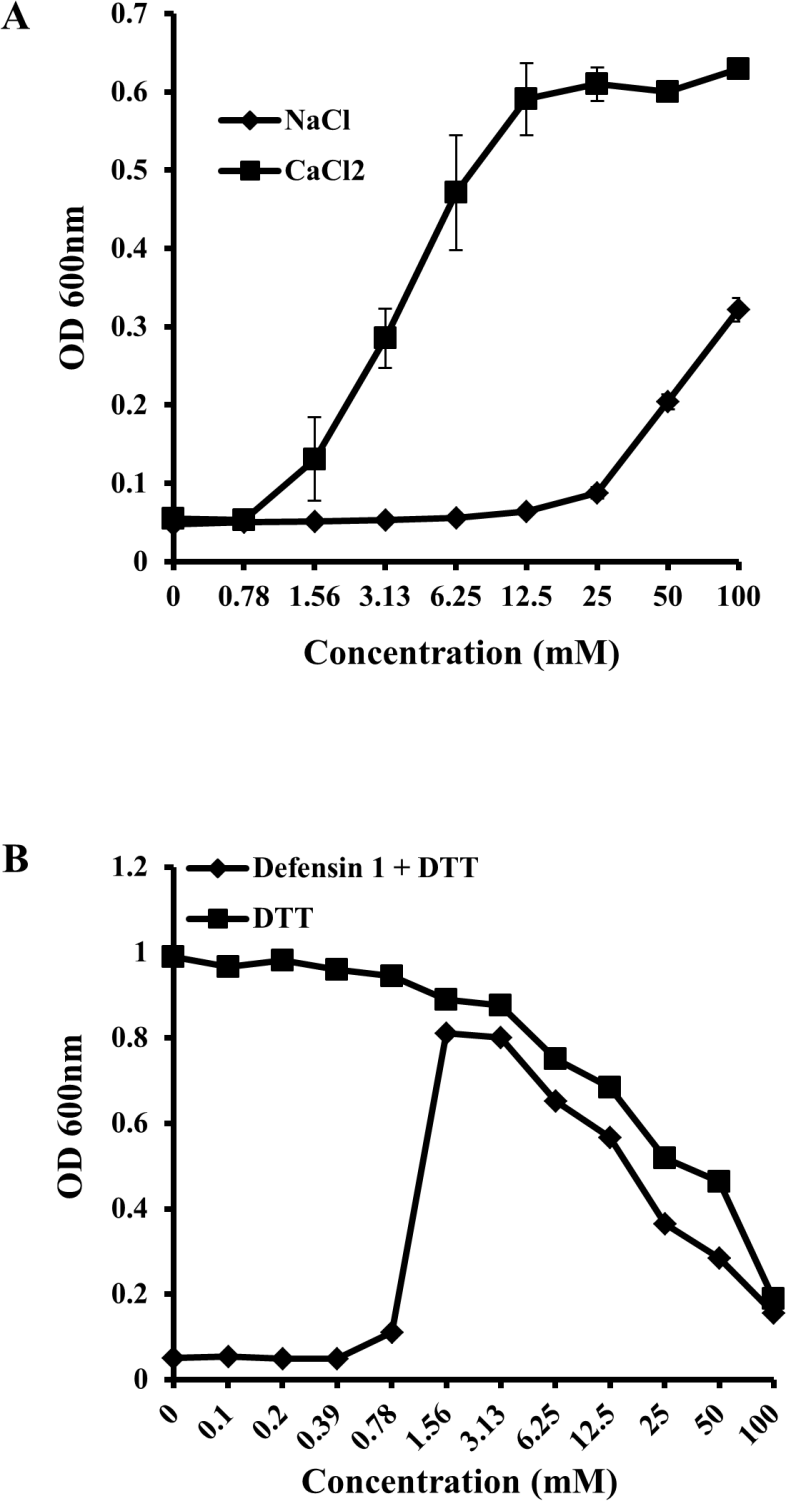
Antimicrobial activity of defensin 1 in the presence of salts and reducing agent. MW2 cells suspended in MH broth were treated with defensin 1 at a constant concentration of 64 μg/ml together with serial dilutions of NaCl or MgCl_2_ **(A)**; or treated with serial dilutions of the reducing agent DTT **(B)**. The OD_600_ was measured after overnight incubation at 35°C.

The sequence of *Tribolium* defensin-1 indicates that it belongs to a typical class of insect defensins, which are composed of cyclic peptides possessing cysteine-stabilized α-helix and β-sheet (CSαβ) structural motifs that are crucial for antimicrobial activity. Its six conserved cysteine residues form intramolecular disulfide bridges (Cys1-Cys4, Cys2-Cys5, Cys3-Cys6) that are important for the folding of the peptide. Secondary structure prediction made using the PSIPRED technique (http://toolkit.tuebingen.mpg.de/quick2_d) suggests that defensin 1 forms β-sheets between the residues 39–43 and 78–80. The secondary structure of mature defensin 1 predicted using the heliquest method (http://heliquest.ipmc.cnrs.fr/cgi-bin/ComputParamsV2.py) suggested that the mature peptide formed an α-helix with a net positive charge similar to other cationic AMPs (Fig 1B). Several cationic AMPs, including peptides of both prokaryotic and eukaryotic origin such as indolicidins, gramicidins, bactenecins, and magainins, are known to be sensitive to salt, which lowers their antimicrobial efficacy [13]. The growth of MRSA strain MW2 was examined in a broth assay in the presence of defensin 1 at a concentration of 64 μg/ml along with serial dilutions of NaCl or MgCl_2_. The final concentratiom of the salts ranged from 0.78 to 100 mM. MgCl_2_ inhibited the antimicrobial activity of defensin 1 even at low concentrations starting at 1.56 mM. NaCl inhibited the activity of defensin 1 starting from a higher concentration of 25 mM (Fig 2A).

The cell envelope of *S*. *aureus* is coated with anionic polysaccharides referred to as wall teichoic acids (WTAs), which play a variety of roles in maintaining bacterial homeostasis [14]. As a form of defense against attack from cationic AMPs, the overall negative charge on the surface of the cell is neutralized by incorporation of D-alanine residues to WTAs, which attenuates the binding of AMPs to the cell envelope. D-alanylation is carried out by the *dlt* operon consisting of the four genes *dltA-D*. Earlier studies have shown that abrogation of D-alanylation through disruption of *dltA*, sensitizes *S*. *aureus* to AMPs [14]. Consistent with the conclusion that defensin 1 functions similarly to previously tested cationic AMPs, we found that the MIC of defensin 1 against the wild type Newman strain was 64 μg/ml whereas the MIC against an isogenic Newman *∆dltA* mutant [15] was 0.5 μg/ml (Table 1).

### Treatment with a reducing agent inhibits the antimicrobial activity of defensin 1

Earlier studies have shown that the antimicrobial properties of AMPs can be inhibited [16] or enhanced [17] by treatment with a reducing agent, such as DTT. In order to study the effect of DTT on the antimicrobial activity of defensin 1, the peptide (final concentration 64 μg/ml) was treated with serial dilutions of DTT adjusted to final concentrations between 0.1 to 100 mM and incubated at room temperature for 1 hour. The effect of DTT alone, native and DTT-treated peptides on the growth of MRSA strain MW2 was examined in a broth assay. DTT by itself displayed a dose dependent growth inhibitory effect on MW2 starting at a concentration of 6.25 mM (Fig 2B). However, at lower concentrations, DTT did not have a direct inhibitory effect on the growth of the bacterium but abolished the antimicrobial activity of defensin 1 at concentrations between 1.56 to 3.13 mM (Fig 2B).”

### Defensin 1 disrupts the *S*. *aureus* cell envelope but does not cause hemolysis of mammalian red blood cells

**Fig 3:**
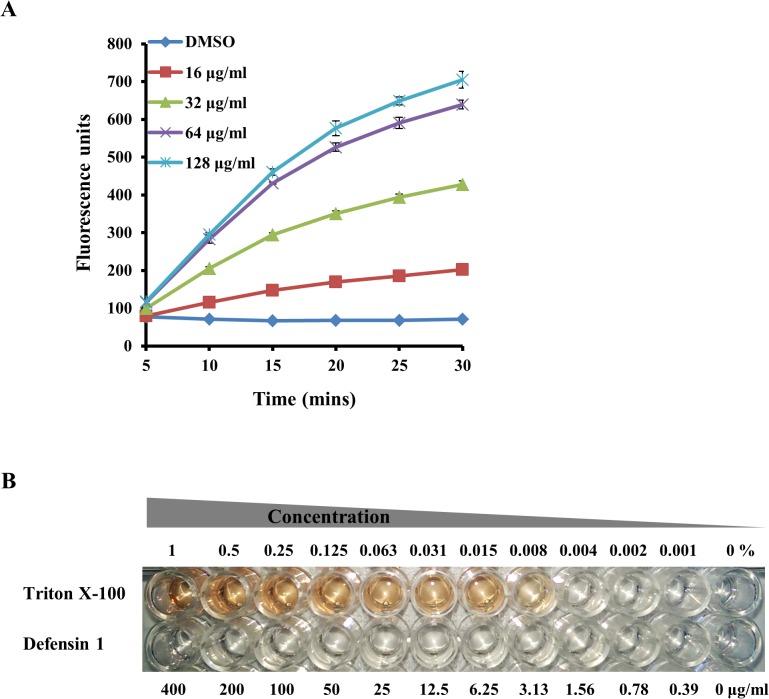
Effect of defensin 1 on bacterial and eukaryotic cell membranes. **(A)** Time course of permeabilization of MRSA strain MW2 cells by defensin 1 was measured by following the increase in Sytox Green fluorescence (λexc = 485 nm; λem = 530 nm). Peptide was tested at serially diluted concentrations between 16 to 128 μg/ml. Cells treated with DMSO (5% final concentration) was the negative control. **(B)** Human erythrocytes were treated with serial dilutions of Triton X-100 (.001–1%) or defensin 1 (0.39–400 μg/ml).

As shown above (Fig 2A), the antibacterial activity of defensin 1 is affected by the overall charge of the cell surface, suggesting that the antibacterial activity of defensin 1 involves physical binding of the AMP to the cell surface. In addition, earlier studies have suggested that several AMPs manifest their antibacterial effect by causing disruptions to the bacterial cell envelope [1]. In order to investigate the antibacterial mode of action of defensin 1, we examined the potential of the peptide to cause disruption of the bacterial cell envelope. MRSA-MW2 cells were exposed to defensin 1 at concentrations between 16–128 μg/ml and bacterial membrane disruption was evaluated by studying uptake of Sytox Green over a period of 30 minutes. Cells treated with defensin 1 displayed a dose dependent uptake of Sytox Green as represented by the increase in cellular fluorescence caused by binding of the dye with the bacterial DNA (Fig 3A). The control cells treated with DMSO showed no change in cellular fluorescence.

The possibility that defensin 1 might induce damage to eukaryotic cell membranes was tested by examining the ability of the peptide to induce hemolysis. Human red blood cells (RBCs) were treated with serial dilutions of defensin 1 (0–400 μg/ml) or triton X-100 (0.001–1% solution) for 1 hour. Defensin 1 did not cause any RBC hemolysis even at the maximum tested concentration of 400 μg/ml (Fig 3B), which was confirmed by measuring absorbance at 540 nm (data not shown). The positive control triton X-100 caused hemolysis at all concentrations starting from 0.008%.

### Defensin 1 displays synergistic activity with telavancin and daptomycin

The results from the earlier section suggest that defensin 1 manifests its antibacterial activity by causing disruption to the bacterial cell envelope. We evaluated the hypothesis that the peptide might be able to function synergistically with clinically utilized antibacterial agents that also target the bacterial cell envelope. Telavancin and daptomycin are two such antibiotics that are clinically effective against drug resistant *S*. *aureus*. Their mode of action involves targetting the bacterial cell membrane [18, 19]. A broth microplate assay was used to test for growth inhibition of MRSA strain MW2, in the presence of defensin 1 adjusted to a final concentration between 1–64 μg/ml, in combination with either telavancin or daptomycin adjusted to final concentrations between 0.016–16 μg/ml. The minimum concentration at which the test compounds inhibited bacterial growth both alone and in combinations were determined to calculate the Fractional Inhibitory Concentration (FIC) index according to the equation shown in the methods section (Table 2). The FIC index was 0.031 and 0.266 for the defensin 1/telavancin and defensin 1/daptomycin combinations, respectively. As the FIC index was less than 0.5 in both cases, this series of experiments suggest that defensin 1 exhibited synergism with both telavancin and daptomycin.

**Table 2:** MIC data for defensin 1, telavancin and daptomycin alone and in combination against MRSA strain MW2.

MIC (μg/ml)				
Defensin 1	Telavancin	Daptomycin	Defensin 1 / Telavancin	Defensin 1 / Daptomycin
64	1	4	1/0.016	1/1
**FIC index**			**0.031**	**0.266**

### Defensin 1 prolongs worm survival in the *C*. *elegans*-MRSA liquid infection assay

**Fig 4:**
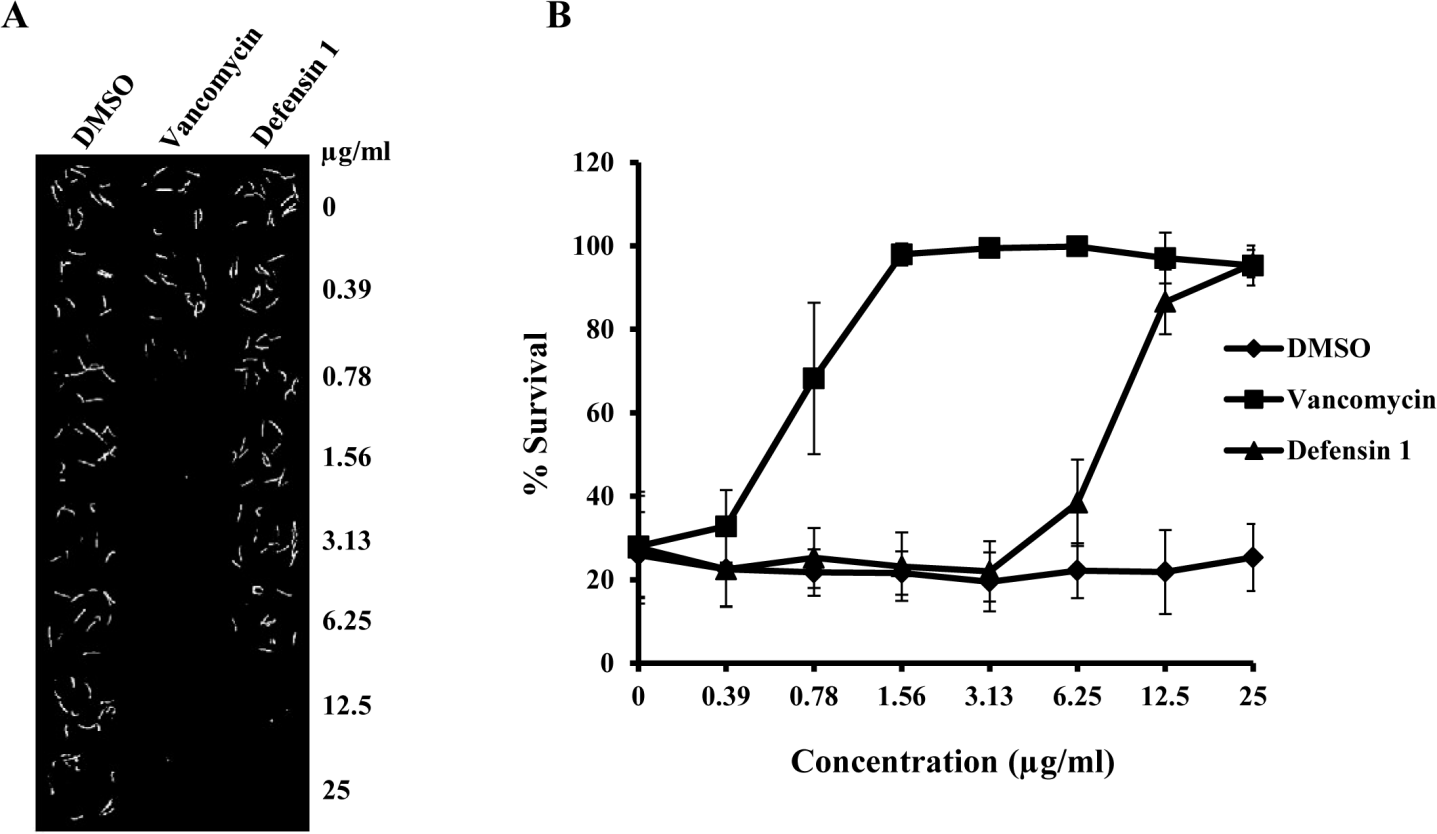
Defensin 1 prolongs survival of *C*. *elegans* infected with MRSA. A 384 well assay plate was co-inoculated with nematodes, bacteria and either DMSO (negative control), vancomycin (positive control) or defensin 1. Serial dilutions of the drugs were tested to adjust the concentration between 0.39–25 μg/ml. **(A)** Sytox Orange stained and bright field images of assay wells containing a gradient of the tested drugs. Dead worms take up the vital dye Sytox Orange and fluoresce. **(B)** Percent survival of infected worms was calculated from intensity of fluorescence measured in each well of the assay plate.

The *C*. *elegans*-MRSA liquid infection assay is an established model for studying toxicity and *in vivo* activity of antimicrobial compounds [5]. The *in vivo* antimicrobial activity of defensin 1 was compared with vancomycin in *C*. *elegans* nematodes infected with MRSA strain MW2. As shown on Fig 4, defensin 1 prolonged survival of infected worms at concentrations starting from 12.5 μg/ml and increased the survival rate of infected worms from 22% to 87% as compared to DMSO treated worms. In the case of vancomycin, starting at a concentration of 1.56 μg/ml, the drug prolonged survival of 98% of the infected worms during the duration of the assay.

## Discussion

Cationic AMPs are a conserved component of the innate immune system of plants and metazoans [1]. The rising incidence of drug resistance in *S*. *aureus* along with the dwindling supply of antibiotics that are effective against these pathogens has led to an interest in AMPs for potential therapeutic applications [1]. The red flour beetle *T*. *castaneum*, a worldwide pest of stored food grains, is also an attractive model to study evolution and immunity in insects due to the availability of the full genome sequence and the ability to perform genetic manipulation, such as RNAi gene knockdowns [4]. Bioinformatics, real-time polymerase chain analysis and suppression subtractive hybridization methods have predicted that *T*. *castaneum* produces 15 AMPs [20]. To our knowledge, the direct antimicrobial effect of these peptides on pathogenic bacteria have not yet been shown. In this study, we have characterized the antimicrobial properties of the *T*. *castaneum* AMP, defensin 1against *S*. *aureus*. Defensin 1 causes disruption of the *S*. *aureus* cell envelope and the antimicrobial activity of the peptide was inhibited by the presence of monovalent or divalent cations. Reduction of the disulfide bonds in defensin 1 by treatment with DTT, inhibited the antimicrobial activity of the peptide. Defensin 1 displayed synergistic activity with other antibiotics that also target the bacterial cell envelope, such as telavancin and daptomycin.

Defensin 1 inhibited growth of *S*. *aureus* at a concentration between 16–64 μg/ml (3.3–13.3 μM) with an average MIC of ~45 μg/ml (9.3 μM). To our knowledge, the physiological concentration levels of defensin peptides in the haemolymph of *T*. *castaneum* have so far not yet been elucidated, but studies with *Aedes aegypti* adult mosquitoes have shown that the concentration of a defensin peptide drastically increased from negligible levels to as high as 45 μM, 24 hours post-challenge with a bacterial inoculum [21]. Similar observations have been made in humans, where the concentrations of host defense peptides are elevated during pathogen insult. The salivary concentration of human neutrophil peptide (HNP-1) is higher in patients suffering from oral inflammation, than in healthy individuals [22].

Many AMPs manifest their antibacterial activity by causing disruptions to the bacterial cell envelope [1] which imparts certain advantages to AMPs as compared to conventional antibiotics that target metabolic pathways. It would be more difficult for bacteria to develop resistance against a compound having a mode of action that is physical in nature. Moreover, even though defensin 1 displayed a high MIC against MRSA, the peptide was able to cause damage to the bacterial cell envelope and also displayed *in vitro* synergism with telavancin and daptomycin, two antibiotics that target the bacterial cell membrane. However, the degree of synergism was more inclined towards telavancin, as compared with daptomycin. Possible reasons for this effect could be the difference in the mode of action of the two antibiotics, even though they both target the cell membrane. It has been shown that telavancin causes physical disruption to the bacterial cell membrane apart from affecting cell wall synthesis, whereas daptomycin aggregates at the cell membrane leading to mislocalization of proteins involved in cell division and cell wall synthesis, which in turn leads to cell death [18, 19]. Combination therapy with antibiotics that also target the bacterial cell membrane could thus be an attractive option for the therapeutic application of defensin 1.

An additional strategy to improve the antibacterial activity of defensin 1 would be to enhance the binding ability of the peptide with the bacterial cell surface, which in turn would enable the peptide to cause more extensive damage to the bacteria. Bacteria have evolved several strategies to attenuate the effect of AMPs, one of which is neutralization of the overall charge of the bacterial cell surface by D-alanine decoration of the wall teichoic acids, which impedes the binding of AMPs to the cell surface [14]. We found that defensin 1 displayed a 128 fold drop in MIC from 64 to 0.5 μg/ml when tested on a mutant strain that was defective in D-alanylation, possibly due to enhanced ability of the peptide to bind to the bacteria. This finding in turn raises the interesting possibility of enhancing antimicrobial activity of defensin 1 against *S*. *aureus* by blocking D-alanylation in the bacteria.

Although the strategies mentioned above might serve to improve the antibacterial activity of defensin 1, an additional factor that needs to be taken into consideration before exploring potential clinical application of defensin 1 is the sensitivity of the peptide to monovalent and divalent cations. In fact, most cationic AMPs are sensitive to high salt concentrations, which act as an impediment to clinical application of cationic AMPs [13]. However, it has been reported that salt resistance in AMPs can be increased by addition of fatty acids, vitamin E, or cholesterol to the termini of AMPs or by replacing tryptophan or histidine residues with the amino acid β-naphthylalanine [13]. It is reasonable to assume that the salt sensitivity of defensin 1 can also be diminished with similar modifications.

To gain further insight into the potential applications of defensin 1, we tested the toxicity of the peptide on human erythrocytes. The absence of any detectable toxicity of defensin 1 against eukaryotic cells also indicates that the peptide should be further investigated in a rodent infection model as a next step. Moreover, the *in vivo* efficacy of the peptide was tested in the *C*. *elegans*-MRSA infection model. Defensin 1 was well tolerated by human erythrocytes and did not cause hemolysis even at the maximum tested concentration of 400 μg/ml. The *C*. *elegans*-*S*. *aureus* infection model has been widely used to study staphylococcal virulence and pathogenesis since key virulence factors that are important for bacterial pathogenesis in the nematode model are also conserved in humans [23]. Defensin 1 was able to prolong survival of *C*. *elegans* infected with MRSA strain MW2 at concentrations starting from 12.5 μg/ml, which is almost 5 fold lower than the *in vitro* MIC against MW2 (64 μg/ml). The minimum effective concentration (MEC) of the peptide *in vivo* being lower than the *in vitro* MIC suggests a possibility that defensin 1 might have immunomodulatory properties. However, the enhanced *in vivo* activity of defensin 1 could also be due to bioaccumulation of the peptide in the infected worms, or indicate a synergistic or additive effect with AMPs produced by the nematode. A recent study done with AMPs produced in bumblebees has shown that co-occuring AMPs are capable of working synergistically and result in more potent antimicrobial effects at low concentrations [24].

Interestingly, defensin 1 demostrated specific activity against *S*. *aureus* and did not display antimicrobial activity against another Gram-positive bacterium such as *Enterococcus faecium* or the Gram-negative bacteria *Klebisella pneumoniae*, *Acinetobacter baumannii*, *Pseudomonas aeruginosa* and *Enterobacter aerogenes*. Obvious reasons for the lack of activity against the Gram-negative bacteria could be due to structural and molecular differences within Gram-positive and Gram-negative cell walls. However, it is unclear why defensin 1 had no effect on the Gram-positive *E*. *faecium*. Similar variability in the activity of cationic peptides against staphylococcal and enterococcal strains have been reported in an earlier study [25]. Additionally, it is also possible that defensin 1 may have a significant degree of specificity to *S*. *sureus*. Synthetic species-specific peptides against *Bacillus subtilis*, *Pseudomonas* spp, *Escherichia coli*, *Klebsiella pneumoniae* and *Salmonella enterica* have been successfully generated in the past [26, 27].

In conclusion, defensin 1 inhibits growth of *S*. *aureu*s by targetting the bacterial cell membrane and displays synergism with antibiotics that also target the bacterial cell membrane. Additionally, the peptide did not exhibit any toxic effect on eukaryotic cells at the tested concentrations. These findings validate the antimicrobial potential of insect AMPs and suggest that their clinical potential should be explored further, especially when combined with actimicrobial agents that downregulate D-alanylation of teichoic acids or with agents that disrupt the staphylococcal cell membrane.

## Supporting Information

S1 Table Sixty five insect AMPs synthesized based on publicly available sequence data and tested for antimicrobial properties against MRSA strain MW2. (XLSX)

## References

[1] Yeung AT, Gellatly SL, Hancock RE. Multifunctional cationic host defence peptides and their clinical applications. Cellular and molecular life sciences: CMLS. 2011;68(13):2161–76. 10.1007/s00018-011-0710-x 21573784 PMC11114888

[2] Hilchie AL, Wuerth K, Hancock RE. Immune modulation by multifaceted cationic host defense (antimicrobial) peptides. Nature chemical biology. 2013;9(12):761–8. 10.1038/nchembio.1393 24231617

[3] Boucher HW, Corey GR. Epidemiology of methicillin-resistant Staphylococcus aureus. Clinical infectious diseases: an official publication of the Infectious Diseases Society of America. 2008;46 Suppl 5:S344–9. 18462089 10.1086/533590

[4] Altincicek B, Knorr E, Vilcinskas A. Beetle immunity: Identification of immune-inducible genes from the model insect Tribolium castaneum. Developmental and comparative immunology. 2008;32(5):585–95. 17981328 10.1016/j.dci.2007.09.005

[5] Rajamuthiah R, Fuchs BB, Jayamani E, Kim Y, Larkins-Ford J, Conery A, et al Whole Animal Automated Platform for Drug Discovery against Multi-Drug Resistant Staphylococcus aureus. PloS one. 2014;9(2):e89189 10.1371/journal.pone.0089189 24586584 PMC3929655

[6] Beanan MJ, Strome S. Characterization of a germ-line proliferation mutation in C. elegans. Development. 1992;116(3):755–66. 1289064 10.1242/dev.116.3.755

[7] Tanaka-Hino M, Sagasti A, Hisamoto N, Kawasaki M, Nakano S, Ninomiya-Tsuji J, et al SEK-1 MAPKK mediates Ca2+ signaling to determine neuronal asymmetric development in Caenorhabditis elegans. EMBO reports. 2002;3(1):56–62. 11751572 10.1093/embo-reports/kvf001 PMC1083920

[8] Poppel AK, Vogel H, Wiesner J, Vilcinskas A. Antimicrobial peptides expressed in medicinal maggots of the blow fly Lucilia sericata show combinatorial activity against bacteria. Antimicrobial agents and chemotherapy. 2015. 10.1128/AAC.05180-14 PMC4394815 25666157

[9] Vilcinskas A, Mukherjee K, Vogel H. Expansion of the antimicrobial peptide repertoire in the invasive ladybird Harmonia axyridis. Proceedings Biological sciences / The Royal Society. 2013;280(1750):20122113 10.1098/rspb.2012.2113 23173204 PMC3574431

[10] Vilcinskas A. Anti-infective therapeutics from the Lepidopteran model host Galleria mellonella. Current pharmaceutical design. 2011;17(13):1240–5. 21470117 10.2174/138161211795703799

[11] Draper LA, Cotter PD, Hill C, Ross RP. The two peptide lantibiotic lacticin 3147 acts synergistically with polymyxin to inhibit Gram negative bacteria. BMC microbiology. 2013;13:212 10.1186/1471-2180-13-212 24069959 PMC3849175

[12] Rosch JW, Boyd AR, Hinojosa E, Pestina T, Hu Y, Persons DA, et al Statins protect against fulminant pneumococcal infection and cytolysin toxicity in a mouse model of sickle cell disease. The Journal of clinical investigation. 2010;120(2):627–35. 10.1172/JCI39843 20093777 PMC2810080

[13] Chu HL, Yu HY, Yip BS, Chih YH, Liang CW, Cheng HT, et al Boosting salt resistance of short antimicrobial peptides. Antimicrobial agents and chemotherapy. 2013;57(8):4050–2. 10.1128/AAC.00252-13 23716061 PMC3719776

[14] Brown S, Santa Maria JP Jr., Walker S. Wall teichoic acids of gram-positive bacteria. Annual review of microbiology. 2013;67:313–36. 10.1146/annurev-micro-092412-155620 24024634 PMC3883102

[15] Santa Maria JP Jr., Sadaka A, Moussa SH, Brown S, Zhang YJ, Rubin EJ, et al Compound-gene interaction mapping reveals distinct roles for Staphylococcus aureus teichoic acids. Proceedings of the National Academy of Sciences of the United States of America. 2014;111(34):12510–5. 10.1073/pnas.1404099111 25104751 PMC4151746

[16] Nuding S, Frasch T, Schaller M, Stange EF, Zabel LT. Synergistic effects of antimicrobial peptides and antibiotics against Clostridium difficile. Antimicrobial agents and chemotherapy. 2014;58(10):5719–25. 10.1128/AAC.02542-14 25022581 PMC4187972

[17] Singh PK, Sharma S, Kumari A, Korpole S. A non-pediocin low molecular weight antimicrobial peptide produced by Pediococcus pentosaceus strain IE-3 shows increased activity under reducing environment. BMC microbiology. 2014;14:226 10.1186/s12866-014-0226-2 25158757 PMC4243815

[18] Lunde CS, Hartouni SR, Janc JW, Mammen M, Humphrey PP, Benton BM. Telavancin disrupts the functional integrity of the bacterial membrane through targeted interaction with the cell wall precursor lipid II. Antimicrobial agents and chemotherapy. 2009;53(8):3375–83. 10.1128/AAC.01710-08 19470513 PMC2715647

[19] Pogliano J, Pogliano N, Silverman JA. Daptomycin-mediated reorganization of membrane architecture causes mislocalization of essential cell division proteins. Journal of bacteriology. 2012;194(17):4494–504. 10.1128/JB.00011-12 22661688 PMC3415520

[20] Ntwasa M, Goto A, Kurata S. Coleopteran antimicrobial peptides: prospects for clinical applications. International journal of microbiology. 2012;2012:101989 10.1155/2012/101989 22500175 PMC3303552

[21] Lowenberger C. Innate immune response of Aedes aegypti. Insect biochemistry and molecular biology. 2001;31(3):219–29. 11167091 10.1016/s0965-1748(00)00141-7

[22] Mizukawa N, Sugiyama K, Ueno T, Mishima K, Takagi S, Sugahara T. Defensin-1, an antimicrobial peptide present in the saliva of patients with oral diseases. Oral diseases. 1999;5(2):139–42. 10522210 10.1111/j.1601-0825.1999.tb00078.x

[23] Sifri CD, Begun J, Ausubel FM, Calderwood SB. Caenorhabditis elegans as a model host for Staphylococcus aureus pathogenesis. Infection and immunity. 2003;71(4):2208–17. 12654843 10.1128/IAI.71.4.2208-2217.2003 PMC152095

[24] Rahnamaeian M, Cytrynska M, Zdybicka-Barabas A, Dobslaff K, Wiesner J, Twyman R, et al. Insect antimicrobial peptides show potentiating functional interactions against Gram-negative bacteria. Proceedings of the Royal Society B. 2015. 10.1098/rspb.2015.0293 PMC4426631 25833860

[25] Friedrich CL, Moyles D, Beveridge TJ, Hancock RE. Antibacterial action of structurally diverse cationic peptides on gram-positive bacteria. Antimicrobial agents and chemotherapy. 2000;44(8):2086–92. 10898680 10.1128/aac.44.8.2086-2092.2000 PMC90018

[26] Eckert R, Qi F, Yarbrough DK, He J, Anderson MH, Shi W. Adding selectivity to antimicrobial peptides: rational design of a multidomain peptide against Pseudomonas spp. Antimicrobial agents and chemotherapy. 2006;50(4):1480–8. 16569868 10.1128/AAC.50.4.1480-1488.2006 PMC1426969

[27] Mondhe M, Chessher A, Goh S, Good L, Stach JE. Species-selective killing of bacteria by antimicrobial peptide-PNAs. PloS one. 2014;9(2):e89082 10.1371/journal.pone.0089082 24558473 PMC3928365

[28] McDougal LK, Steward CD, Killgore GE, Chaitram JM, McAllister SK, Tenover FC. Pulsed-field gel electrophoresis typing of oxacillin-resistant Staphylococcus aureus isolates from the United States: establishing a national database. Journal of clinical microbiology. 2003;41(11):5113–20. 14605147 10.1128/JCM.41.11.5113-5120.2003 PMC262524

[29] Nair D, Memmi G, Hernandez D, Bard J, Beaume M, Gill S, et al Whole-genome sequencing of Staphylococcus aureus strain RN4220, a key laboratory strain used in virulence research, identifies mutations that affect not only virulence factors but also the fitness of the strain. Journal of bacteriology. 2011;193(9):2332–5. 10.1128/JB.00027-11 21378186 PMC3133102

[30] Baba T, Bae T, Schneewind O, Takeuchi F, Hiramatsu K. Genome sequence of Staphylococcus aureus strain Newman and comparative analysis of staphylococcal genomes: polymorphism and evolution of two major pathogenicity islands. Journal of bacteriology. 2008;190(1):300–10. 17951380 10.1128/JB.01000-07 PMC2223734

[31] Mack D, Nedelmann M, Krokotsch A, Schwarzkopf A, Heesemann J, Laufs R. Characterization of transposon mutants of biofilm-producing Staphylococcus epidermidis impaired in the accumulative phase of biofilm production: genetic identification of a hexosamine-containing polysaccharide intercellular adhesin. Infection and immunity. 1994;62(8):3244–53. 8039894 10.1128/iai.62.8.3244-3253.1994 PMC302952

